# ﻿*Ixeridiumnujiangense* (Crepidinae, Cichorieae, Asteraceae), a new species from southwest Yunnan, China

**DOI:** 10.3897/phytokeys.244.126940

**Published:** 2024-07-11

**Authors:** Ze-Huan Wang, Qian-Qian Zhong, Yong-Liang Li, Jia-Ju Xu, Qing-Wen Sun

**Affiliations:** 1 Department of Traditional Chinese Medicine Resources and Development, College of Pharmacy, Guizhou University of Traditional Chinese Medicine, Guiyang 550025, Guizhou, China Guizhou University of Traditional Chinese Medicine Guiyang China; 2 Administration Bureau of Yongde Daxueshan National Nature Reserve, Yongde 677600, Yunnan, China Administration Bureau of Yongde Daxueshan National Nature Reserve Yongde China

**Keywords:** *Ixeridiummalingheense*, morphology, new species, Nujiang River, taxonomy

## Abstract

In this paper, we describe *Ixeridiumnujiangense*, a novel species identified in southwestern Yunnan, China. Two populations have been found along the riverbanks of the Nujiang River in Yongde and Zhenkang Counties. Morphologically, *I.nujiangense* is most similar to the recently described *I.malingheense*, but it can be readily distinguished by its mostly divided basal leaves, narrower non-clasping cauline leaves, notably shorter corolla tube, pale brown anthers, and considerably longer beak of achenes.

## ﻿Introduction

The Asteraceae, recognized as the most species-rich plant family, continues to draw scholarly attention with reports of new genera and species discovered within its ranks. Over the past five years, five new genera have been identified in China: *Sinoseris* N.Kilian, Ze H.Wang & H.Peng (Wang et al. 2020), *Lipschitzia* Zaika, Sukhor. & N.Kilian (Zaika et al. 2020), *Lihengia* Y.S.Chen & R.Ke (Chen et al. 2021), *Mojiangia* Ze H.Wang, N.Kilian et H.Peng (Yin et al. 2022), and *Qineryangia* Y.S.Chen et L.S.Xu (Xu LS et al. 2024). In addition, numerous new species from various genera have been reported (Wang et al. 2020; Liu et al. 2021; Lu et al. 2021; Xiao et al. 2022; Yin et al. 2022; Jin et al. 2023; Zhong et al. 2023; Xu JJ et al. 2024; Xu LS et al. 2024; Xu Q et al. 2024), highlighting that the exploration and documentation of the Asteraceae species are far from complete.

*Ixeridium* (A.Gray) Tzvelev is a moderately-sized genus in the Crepidinae subtribe of the Asteraceae family. The significant morphological variations, along with overlapping distributions, create challenging groups that are hard to differentiate. The most typical example is the *I.dentatum* complex. Tanaka and Takahara (2013) conducted a comprehensive and detailed study on this complex, redefined the taxonomic ranks of various groups, and resolved the long-standing classification issues of this complex. The taxonomy of *Ixeridium* species in China has also undergone significant changes in recent years. First, during the compilation of the Flora of China, Shih and Kilian (2011) discovered that *I.beauverdianum* (H.Lév.) Springate had been misidentified as *I.gracile* (DC.) Pak & Kawano in China and revised the classification accordingly. Secondly, research by Zhang et al. (2023) revealed that *I.sagittarioides* (C.B.Clarke) Pak & Kawano should belong to the genus *Lactuca* L., hence requiring its removal from *Ixeridium*. Additionally, the newly described species *I.calcicola* C.-I.Peng, S.W.Chung & T.C.Hsu (Nakamura et al. 2014), *I.dimorphifolium* Y.L.Xu, Y.F.Lu & X.Cai (Lu et al. 2021), and *I.malingheense* Z.Li & Q.Xu (Xu Q et al. 2024) have expanded our understanding of the morphological diversity within this genus.

Through the continuous efforts of taxonomists, the species range and systematic relationships within *Ixeridium* are becoming increasingly clear. Recent studies have established that the genus *Ixeridium* comprises 17 species, predominantly distributed across East and Southeast Asia. Of these, ten species are native to China, with six being endemic (Shih and Kilian 2011; Nakamura et al. 2014; Lu et al. 2021; Zhang et al. 2023; Xu Q et al. 2024). The plants of this genus are characterized by yellow ligulate florets, somewhat compressed achenes with a slender beak, and a typically yellowish to straw-colored pappus (Shih and Kilian 2011). Although *Ixeridium* is morphologically similar to *Ixeris* (Cass.) Cass., it can be distinguished by its achenes, which have 9–12 prominent, non-wing-like ribs, vs. the wing-like, 10-ribbed achenes of *Ixeris* (Shih 1997).

In December 2023, while collecting plant seeds in Banlao Village, Xiaomengtong Town, Yongde County, the authors discovered a plant with slender leaf petioles on the exposed riverbank of the Nujiang River. Upon returning to the site in February 2024, further observations were made regarding the plant’s floral and fruit morphology. On the same day, another population was found along the Nujiang River in Yakou Village, Mengpeng Town, Zhenkang County, adjacent to Yongde County. Detailed examination of these specimens confirmed that they belong to a previously undescribed species, which we present and describe herein.

## ﻿Materials and methods

### ﻿Morphological analysis

To characterize the morphology of the newly discovered species, we conducted on-site observations and captured photographs of the plants in their natural surroundings. Additionally, we analyzed herbarium specimens sourced from these locations (KUN, GTZM). To facilitate a comparative morphological study, we referred to the taxonomic keys provided in the Flora Reipublicae Popularis Sinicae (Shih 1997) and the Flora of China (Shih and Kilian 2011), in conjunction with examining the original descriptions and types of *I.yunnanense* C.Shih and the three new Chinese species of *Ixeridium* (Nakamura et al. 2014; Lu et al. 2021; Xu Q et al. 2024). Our examination of the achenes and pappus involved the use of an anatomical microscope (SDPTOP OD500H), while the lengths of ligules, anther tubes, and achenes were measured using a light microscope (Olympus DP72) on both fresh and preserved specimens.

### ﻿Taxon sampling and outgroup selection

A phylogenetic analysis was conducted to determine the systematic position of the new species within the genus *Ixeridium*. Sequencing of the new species was carried out along with *I.yunnanense* and *I.malingheense*. The analysis was primarily based on the established framework of *Ixeridium* outlined in Nakamura et al. (2014) and Xu Q et al. (2024). When multiple sequences were available for species of *Ixeridium*, two sequences were randomly selected to represent their systematic positions. A total of 24 sequences representing 15 species (including 6 subspecies) of *Ixeridium* were included in the phylogenetic analysis. Additionally, 6 sequences from 6 species of the sister genera *Ixeris* were included to assess their relationships. To maintain consistency, *Youngiajaponica* (L.) DC., *Crepidiastrumlanceolatum* (Houtt.) Nakai, and *Paraixerisdenticulata* (Houtt.) Nakai were used as outgroups. The species names in all sequences are followed by GenBank accession numbers for easy identification of their sources. Information on the newly sampled taxa, along with their voucher details and GenBank accessions, is provided in Table 1.

**Table 1.** Information on the newly sampled taxa, along with their voucher details, and GenBank accessions.

Sample name	Locality	Collector & Collection no.	Genbank accession
*Ixeridiumnujiangense*	China, Yunnan, Yongde, Xiaomengtong, Banlao, along the bank of Nujiang River, alt. 541 m	Wang Zehuan & Li Yongliang wzh20240201	PP892766
	China, Yunnan, Zhenkang, Mengpeng, Yakou, along the bank of Nujiang River, alt. 537 m	Wang Zehuan & Li Yongliang wzh20240202	PP892767
*Ixeridiummalingheense*	China, Guizhou, Xingyi, Zhaojiadu, along the bank of Malinghe Canyon, alt. 833 m	Wang Zehuan et al. wzh20240301	PP906177
*Ixeridiumyunnanense*	China, Yunnan, Yongde, Wumulong, on the slope of Yanglang River, alt. 1860 m	Li Yongliang 20240601	PP906175
			PP906176

### ﻿DNA extraction and PCR amplification

We selected the nuclear ribosomal internal transcribed spacer (nrITS), a commonly used barcoding fragment, to explore the phylogenetic relationship between the new species and other *Ixeridium* species. The modified CTAB method was employed to extract DNA from the samples. Universal primers ITS4 and ITS5 (White et al. 1990) were used for PCR amplification. The PCR mixture employed in the study was procured from Sangon Biotech Company (China), and the recommended PCR cycling conditions for this mixture (95 °C for 3 min, 30 cycles of 95 °C for 30 s, 55 °C for 30 s, 72 °C for 30 s, with a final extension at 72 °C for 5 min) were applied for amplification. Subsequently, gel electrophoresis was conducted, and the eligible samples were forwarded to Sangon Biotech Company, China, for sequencing.

### ﻿Phylogenetic reconstruction

The sequencing quality and chromatograms of the newly acquired five sequences were assessed with Bioedit v.7.0 (Hall 1999). These sequences were aligned with others from GenBank via Muscle (Edgar 2004), followed by manual adjustment and trimming in PhyDE v.0.9971 (Müller et al. 2010). The resulting .fas file was then transferred to Phylosuite v.1.2.3 (Xiang et al. 2023) for analysis. Phylogenetic relationships were reconstructed through Maximum Likelihood (ML) and Bayesian Inference (BI) methods. Models were selected by ModelFinder in Phylosuite with default parameters based on the Akaike Information Criterion (AIC). The ML tree was constructed with IQ-TREE in Phylosuite, applying the selected IQ-TREE model and 1,000 bootstrap replicates for branch support. The BI tree was generated through MrBayes in Phylosuite, using the selected MrBayes model and running for 5,000,000 generations with default parameters. Finally, both trees were visualized and edited with FigTree v.1.4.4 (Rambaut 2018).

We defined branches with posterior probabilities (PP) < 0.70 and bootstrap values (BS) < 60 as weakly supported, 0.70 ≤ PP < 0.95 and 60 ≤ BS < 80 as moderately supported, and PP ≥ 0.95 and BS ≥ 80 as strongly supported.

## ﻿Results

### ﻿Morphological studies

**Figure 1.**
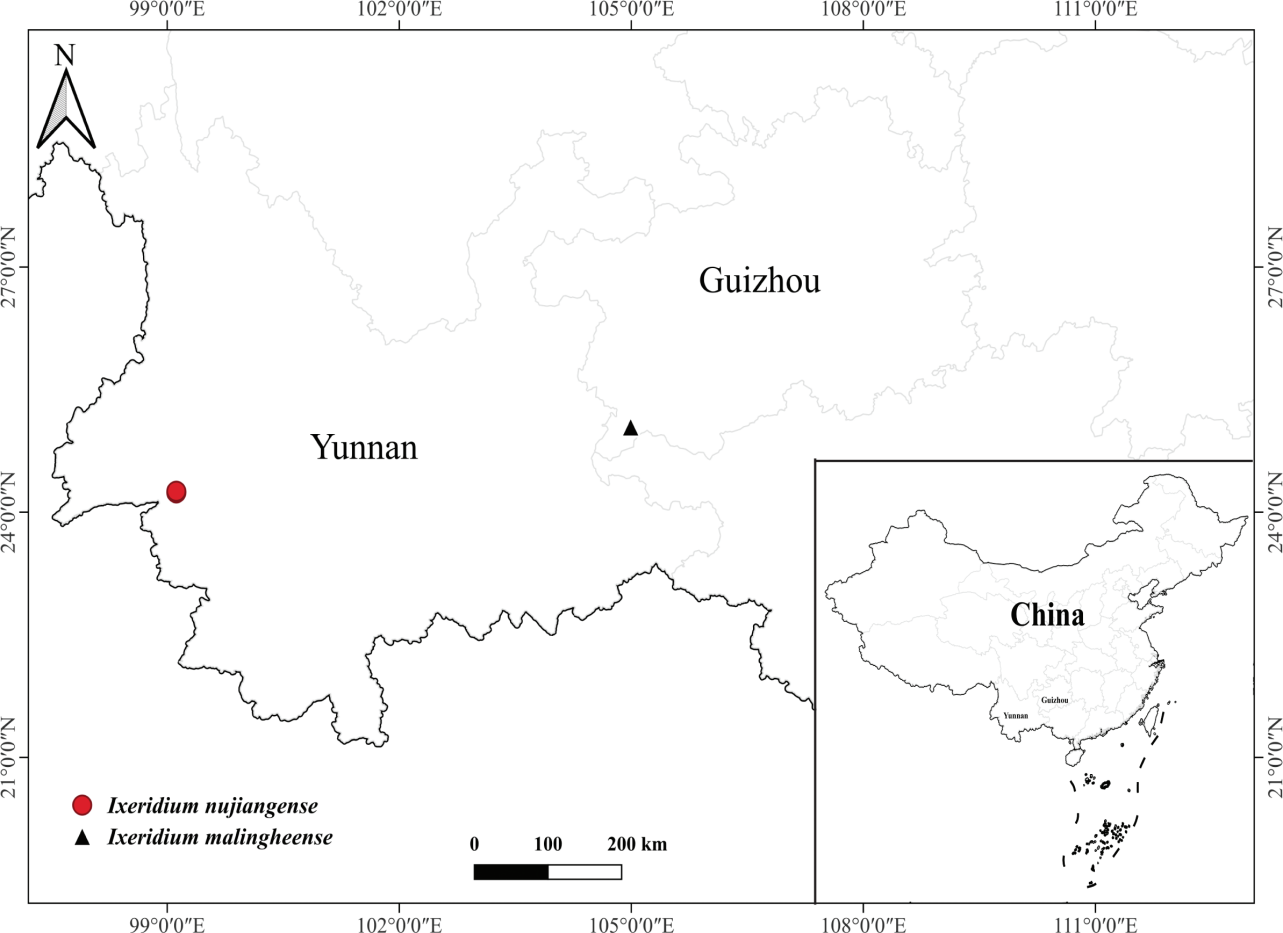
Distribution map of *Ixeridiumnujiangense* and *I.malingheense*.

**Figure 2.**
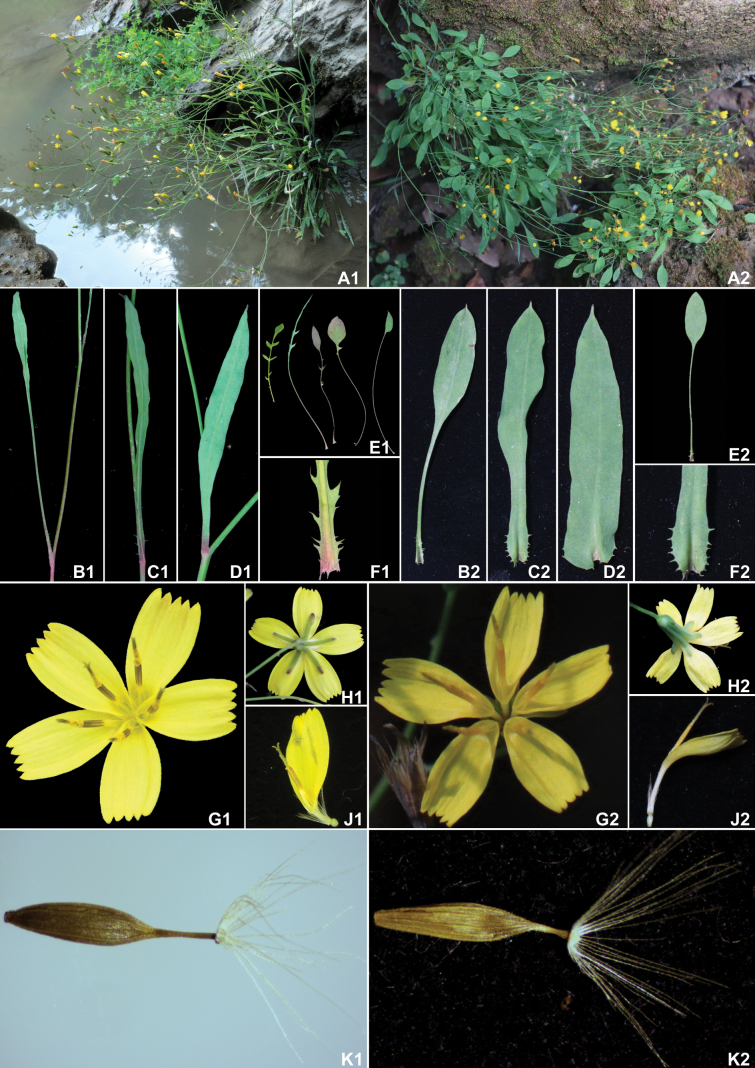
Comparison of the morphological characteristics between *Ixeridiumnujiangense* (A1–K1) and *I.malingheense* (A2-K2). **A** plants **B** lower cauline leaves **C** middle cauline leaves **D** upper cauline leaves **E** basal leaves **F** base of middle cauline leaves **G** capitula in front view **H** capitula in back view **J** florets **K** achenes.

After detailed morphological studies, we found that among all currently published and accepted species of the genus *Ixeridium*, *I.nujiangense* is morphologically most similar to the recently described *I.malingheense*. Although the two species have different distribution locations (Fig. 1), they both grow along river banks and have an early flowering period, usually in winter and spring. Morphologically, they both possess 5 inner phyllaries and relatively few ligulate florets (typically 5–6), with similar corolla and achene lengths. However, the two species exhibit significant differences in the morphology of their basal and cauline leaves, anther color, and the length of the achene beak (Fig. 2). The main morphological differences between the two species are detailed in Table 2.

**Table 2.** Comparison of the morphological characteristics between *Ixeridiumnujiangense* and *I.malingheense*.

Characteristics	*I.nujiangense*	*I.malingheense*
**Basal leaves**	usually pinnatisect or pinnately divided, with only a few entire leaves	all entire
**Cauline leaves**	lanceolate to linear-lanceolate, 0.1–0.3 cm wide, with upper leaf base attenuate, not clasping the stem	oblong or narrowly elliptical, 0.2–0.9 cm wide, with upper leaf base auriculately clasping the stem
**Phyllary**	apex purplish-brown, inner phyllaries 4.8–5.9 mm in length	apex green, inner phyllaries 4.1–5.3 mm in length
**Corolla**	5.9–8.1 mm in length	5.1–6.2 mm in length
**Corolla tube**	ca. 1/5 of the corolla length, pale yellow	ca. 1/3 of the corolla length, white
**Anther**	pale brown	yellow
**Style**	apex ca. 2/3 of the ligule	apex equals the ligule
**Achene**	2.9–3.1 mm in length	2.5–2.6 mm in length
**Beak**	0.9–1.1 mm long, ca. 1/3 of the achene length	0.5–0.8 mm long, ca. 1/6 of the achene length

### ﻿Phylogenetic analysis

**Figure 3.**
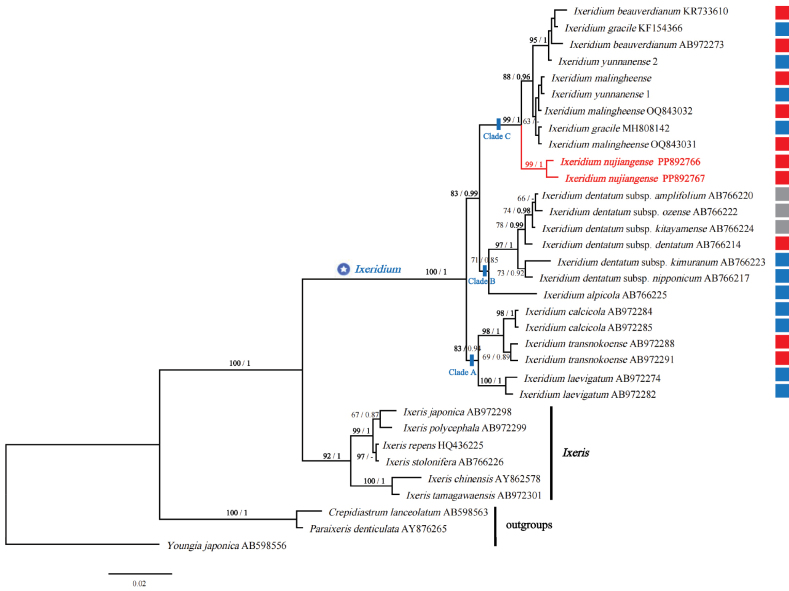
The consensus phylogenetic tree derived from Bayesian Inference (BI) analysis based on nrITS sequence data. Bootstrap support values from Maximum Likelihood (ML) analysis (ML > 60, left) and Bayesian posterior probabilities (PP > 0.70, right) are indicated above the branches. Strongly supported values (BS > 80, PP > 0.95) are shown in bold. Small boxes represent capitulum morphology: red indicates inner phyllaries ca. 5 and ligulate florets usually 5–6, blue indicates inner phyllaries ca. 8 and ligulate florets 8–12, and gray indicates uncertain characteristics.

The matrix used for the final phylogenetic analysis includes a total of 33 sequences, their final aligned matrix is 643bp long, with 126 informative sites. Using the Akaike Information Criterion (AIC), ModelFinder selected the SYM+G4 model for both IQ-Tree and MrBayes. The Maximum Likelihood (ML) and Bayesian Inference (BI) phylogenetic tree constructed based on this model exhibit almost the same topology. The BI consensus tree, including both bootstrap support (BS) and posterior probabilities (PP) values, is shown in Fig. 3.

The phylogenetic tree shows that all sequences of *Ixeridium* cluster into a single clade with strong support (BS=100, PP=1). Within this clade, there are three subclades with moderate or strong support: clade A (BS=83, PP=0.94), clade B (BS=71, PP=0.85), and clade C (BS=99, PP=1). The two sequences of the new species *I.nujiangense* from two distribution points cluster into a strongly supported small subclade (BS=99, PP=1), forming the basal branch of clade C, and sister to a subclade (BS=88, PP=0.96) formed by *I.malingheense*, *I.yunnanense*, *I.gracile*, and *I.beauverdianum*.

### ﻿Taxonomy

#### ﻿ Ixeridium nujiangense

Taxon classificationPlantae

Ze H.Wang sp. nov.

68517D9C-E5AA-5F0C-9BE0-BD41B70D9F4D

urn:lsid:ipni.org:names:77345032-1

Figs 2 , 4

##### Type.

China, Yunnan Province, Lincang City, Yongde County, Xiaomengtong Town, Banlao Village, along the bank of Nujiang River, 24°15'19.70"N, 99°07'03.02"E, alt. 541 m, 25 Feb 2024, *Wang Zehuan & Li Yongliang wzh20240201* (holotype: KUN!, isotypes: KUN!, GTZM!).

##### Diagnosis.

*Ixeridiumnujiangense* is morphologically most similar to *I.malingheense* but can be distinguished by the following traits: basal leaves usually pinnatisect or pinnately divided, with only a few entire leaves (vs. basal leaves all entire), cauline leaves lanceolate to linear-lanceolate, with upper leaf base attenuated, not clasping (vs. cauline leaves oblong or narrowly elliptical, with upper leaf base auriculately clasping), corolla tube ca. 1/5 of the corolla length (vs. corolla tube ca. 1/3 of the corolla length), anthers pale brown (vs. anthers yellow), achenes 2.9–3.1 mm in length (vs. achenes 2.5–2.6 mm in length), beak ca. 1/3 of the achene length (vs. beak ca. 1/6 of the achene length).

##### Description.

Perennial herbs, 17–37 cm tall, glabrous totally, with white latex. Roots fibrous and densely shoot-bearing, fleshy, up to 0.6 cm in diameter. Stems erect, slender, often branched near the base or below the middle. Leaves glabrous, green on the upper surface and pale green on the lower surface, margin entire or with sparsely slender teeth. Basal leaves rosulate, persistent at anthesis, blades 1.5–5.5 × 0.5–1 cm, entire, pinnatisect or pinnately divided, petioles 6.2–7.5 cm long. Terminal lobes of divided leaves 1.1–3 × 0.3–0.6 cm, ovate, elliptic to narrowly lanceolate, apex obtuse, acute to acuminate, base slightly attenuate; lateral lobes 1–4, concentrated at the lower and middle portions of the leaf, the lower lobes smaller and serrate, the middle lobes larger, elongated or obliquely triangular, gradually tapering towards the acuminate apex. Cauline leaves 3–4, blades lanceolate to linear-lanceolate, 1.3–9.4 × 0.1–0.3 cm, apex acute to acuminate, base attenuate, margin entire and with/without sparsely slender ciliate teeth or serrate lobes at the base. Synflorescence corymbiform, with numerous capitula; capitula with 5(–6) florets, base with slender, long peduncle. Bracts linear-lanceolate, up to 7 mm long. Involucre narrowly cylindrical, ca. 5–6 mm long. Phyllaries in two series, glabrous; outer phyllaries broadly ovate, 0.3–0.5 × 0.5 mm, apex obtuse and purplish-brown; inner phyllaries 5, linear-lanceolate, 4.8–5.9 × 0.5–0.9 mm, green on both sides, with transparent membranous margins, apex obtuse and purplish-brown. Receptacle flattened, glabrous, alveolate. Florets 5(–6), ligulate, yellow, corolla 5.9–8.1 mm long, tube 1.0–1.9 mm long, pale yellow, ligules ca. 4.9–6.2 × 1.5–2.1 mm; anthers brown, anther tube 1.8–1.9 mm long; ovary ellipsoid, style ca. 5 mm long. All achenes uniform in shape, pale brown, narrowly fusiform, slightly compressed, 2.9–3.1 mm long, with 3 fine ribs on each side, apex attenuate to a slender beak approximately 0.9–1.1 mm in length. Pappus straw-colored, ca. 3 mm long, 1-seriate, scabrid.

**Figure 4.**
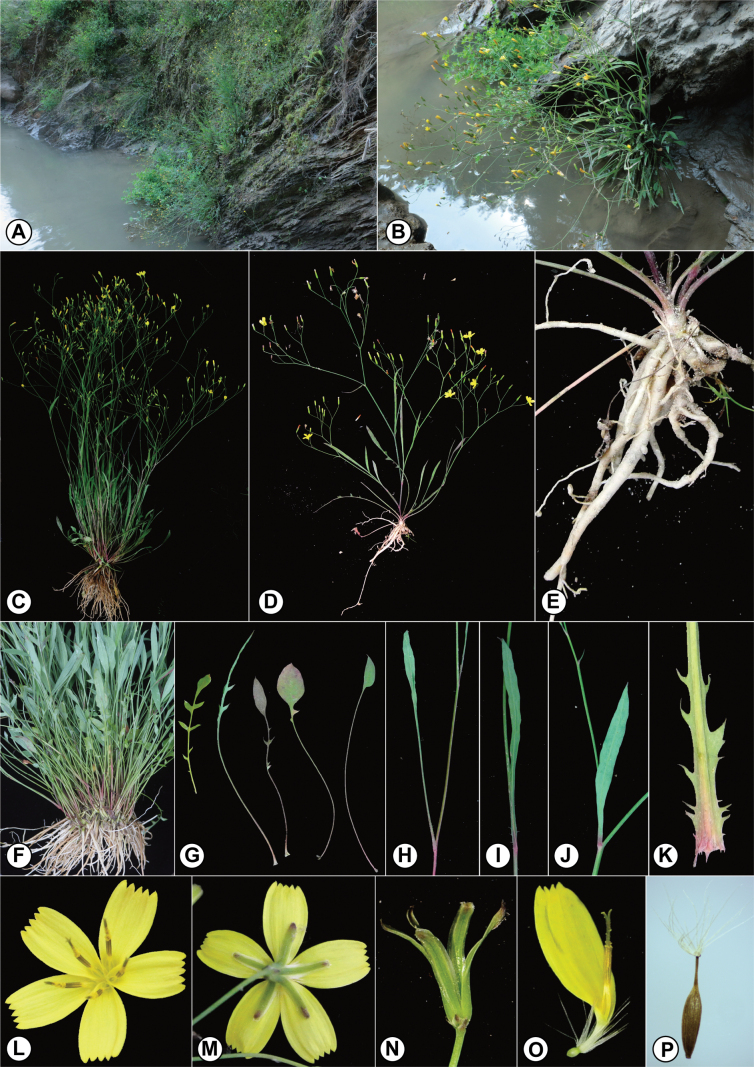
*Ixeridiumnujiangense* sp. nov. **A**–**B** habitat **C**–**D** plants **E** fleshy root **F** intertwined roots and basal leaves **G** basal leaves **H** lower cauline leaf **I** middle cauline leaf **J** upper cauline leaf **K** lower part of middle cauline leaf **L** capitulum in front view **M** capitulum in back view **N** involucre **O** floret **P** achene with pappus.

##### Distribution and habitat.

*Ixeridiumnujiangense* is currently known from two locations in Yongde and Zhenkang Counties, Yunnan, China. This species thrives on the banks of the Nujiang River, which are exposed during the dry season, at an elevation of ca. 540 m. *Lotuscorniculatus* L. (Fabaceae) is the primary associated species, sharing the barely vegetated riverbank terrain with *I.nujiangense*.

##### Phenology.

Flowering and fruiting occur from December to March.

##### Etymology.

The specific epithet ‘*nujiangense*’ is derived from the name of the Nujang River along whose banks the type locality is situated.

##### Vernacular name.

Simplified Chinese:怒江小苦荬; Chinese Pinyin: Nùjiang Xiǎokǔmǎi.

##### Additional specimens examined.

China, Yunnan Province, Lincang City, Zhenkang County, Mengpeng Town, Yakou Village, along the bank of Nujiang River, 24°14'08.11"N, 99°07'03.02"E, alt. 537 m, 25 Feb 2024, Wang Zehuan & Li Yongliang wzh20240202 (KUN!, GTZM!).

## ﻿Discussion

In this study, we sampled all Chinese *Ixeridium* species, except for the doubtful species *I.aculeolatum* C.Shih and the newly described species *I.dimorphifolium* (Lu et al. 2021), to explore their phylogenetic relationships. From the ML and BI phylogenetic trees, it can be seen that the genus *Ixeridium* is monophyletic and can be divided into three clades. The new species *I.nujiangense* is located in Clade C and forms a sister group with a subclade that includes the recently described *I.malingheense*, as well as *I.beauverdianum*, *I.gracile*, and *I.yunnanense*.

Two types of capitula can generally be classified based on the morphology of Chinese *Ixeridium* species: Type One, usually has 5 inner phyllaries and often 5–6 ligulate florets, and Type Two, commonly has 8 inner phyllaries and 8–12 ligulate florets. However, all three evolutionary lineages within the genus *Ixeridium* contain species exhibiting both types of capitula (Fig. 3). This indicates that the easily discernible morphological characteristics of the capitula do not correspond to the evolutionary relationships among species within the genus *Ixeridium*.

Additionally, within Clade C, the two sequences of the new species *I.nujiangense* cluster closely together (BS=99, PP=1). Notably, one sequence each from *I.gracile* and *I.yunnanense* clusters with *I.beauverdianum*, while their other sequences cluster with *I.malingheense*. Upon careful examination of the sequence mutations, researchers observed that the sequences of *I.gracile* and *I.yunnanense*, which are grouped with *I.beauverdianum*, do indeed exclusively share two informative mutational sites with it. These shared mutations likely play a key role in forming a well-supported branch for these four sequences (BS=95, PP=1). To better differentiate these five species, an identification key is provided as follows.

### ﻿Key to the five species of Clade C

**Table:** 

1a	Inner phyllaries 7–8; florets 8–12; achene ca. 5–6 mm in length	**2a**
2a	Involucre 5–6 mm; basal leaves elliptic or obovate, sometimes lanceolate or oblanceolate, 2–5 × 0.5–1.2 cm	***Ixeridiumyunnanense***
2b	Involucre 7–8 mm; basal leaves narrowly spatulate, narrowly elliptic, or almost linear, 4–15 × 0.4–1 cm	***Ixeridiumgracile***
1b	Inner phyllaries 5; florets 5–6(–7); achene less than 4 mm in length	**3a**
3a	Basal leaves usually pinnatisect or pinnately divided, with only a few entire leaves; beak ca. 1/3 of the achene length	***Ixeridiumnujiangense***
3b	Basal leaves entire or with a few very slender linear teeth; beak ca. 1/6 of the achene length	**4a**
4a	Basal leaves long-spatulate or spatulate; anthers yellow; achenes 2.5–2.6 mm in length	***Ixeridiummalingheense***
4b	Basal leaves narrowly elliptic to linear; anthers brown; achenes 3.2–3.5 mm in length	***Ixeridiumbeauverdianum***

## Supplementary Material

XML Treatment for Ixeridium nujiangense

## ﻿Additional information

### Author ORCIDs

Ze-Huan Wang https://orcid.org/0000-0002-7369-5005

Qian-Qian Zhong https://orcid.org/0009-0002-7051-1503

Yong-Liang Li https://orcid.org/0009-0004-5230-2921

Jia-Ju Xu https://orcid.org/0009-0004-2852-0611

Qing-Wen Sun https://orcid.org/0000-0003-0848-5017

### Data availability

All of the data that support the findings of this study are available in the main text.
